# The Effect of Salt Reduction and Partial Substitution of NaCl by KCl on Physicochemical, Microbiological, and Sensorial Characteristics and Consumers' Acceptability of Semi-Hard and Hard Lactose-Free Cow's Milk Cheeses

**DOI:** 10.3389/fnut.2022.861383

**Published:** 2022-05-02

**Authors:** Bibiana Juan, Antonio-José Trujillo, Victoria Ferragut

**Affiliations:** Centre d'Innovació, Recerca i Transferència en Tecnologia dels Aliments (CIRTTA), XaRTA, TECNIO, MALTA-Consolider, Departament de Ciència Animal i dels Aliments, Facultat de Veterinària, Universitat Autònoma de Barcelona (Cerdanyola del Vallès), Barcelona, Spain

**Keywords:** sodium reduction, sodium substitution, potassium, yeast extract, biochemical composition, cheese microbiota, sensorial quality

## Abstract

Increasing consumer demand for healthier foods prompts the development of cheeses reduced in salt. The aim of his study was to assess the effect of reducing the level of sodium chloride (NaCl) and their partial substitution by potassium chloride (KCl) on the biochemical, microbiological, and sensorial characteristics and consumer's acceptability of semi-hard and hard lactose-free cow's milk cheeses. To improve the possible lower salty taste or the development of bitter taste, the addition to yeast extract, as a flavor enhancer, was also checked. Different brining times and brine conditions were tested to obtain a reduction of >25% of salt with respect to conventional cheese. Reduced-salt cheeses were elaborated by reducing half the salting time used in conventional cheeses, and a ratio of 60 Na^+^:40 K^+^ was used to reduce Na concentration in substituted cheeses. The results obtained in this study show that the reduction of salt by both methods in semi-hard and hard lactose-free cheeses could be an alternative for the production of healthier and sensorial acceptable cheeses, without significantly affecting their physicochemical characteristics. The addition of yeast extract, as a flavor enhancer, increased the free amino acids (FAAs) levels but decreased the acceptability of cheeses.

## Introduction

Cheese is one of the most diverse varieties of dairy products and has become an important contributor to nutritional intake in public health interest because it supplies indispensable nutrients for human nutrition, especially proteins, bioactive peptides, lipids, vitamins, and minerals, essentially calcium, magnesium, and phosphorus (1). However, the high sodium (Na) levels in cheese are of major concern for public health worldwide, as it has been associated with a high risk of hypertension, cardiovascular disease, and other chronic non-communicable diseases (2). The World Health Organization (WHO) has recommended to food manufacturers to minimize the Na salt content in their products, particularly in the main food contributors to dietary salt intake, in which recommendation of a daily intake is <2 g Na per day [5 g salt per day (3)]. In general, cheese and dairy products are listed as the third-largest contributor of salt intake and are reported to contribute around 10% of the Na in most European countries (4).

At present, consumers are more aware of the relationship between food and health and are demanding healthier foods. Therefore, the dairy industry is continuously looking for alternative ways to reduce the salt content of cheese while maintaining consumer satisfaction. This task is not easy because salt plays an important role on cheese production, affecting the technological and sensorial aspects of the final product. In addition to providing flavor, salt controls microbial growth, enzyme activity, and curd syneresis, which in turn affects cheese composition (5, 6). In addition, salt can affect proteolysis behavior, which may be related to sensory defects, including the development of bitterness and reduction of firmness (7, 8).

Several strategies for reducing the salt levels in cheeses have been investigated (7–9). The simple reduction of sodium chloride (NaCl) is one of them and can be achieved by reducing brining time (10), using brine with different salt concentrations (11), or adding less salt to cheese curd (9). Diverse studies have been carried out to investigate the lowest salt concentration that can be applied without affecting cheese quality. In general, salt-free cheeses demonstrate undesirable sensory characteristics when compared to cheeses with intermediate and conventional levels of salt (7, 8, 12–14).

Another common technique is the use of salt substitutes to reduce the amount of Na. Different mineral salt replacers, such as potassium chloride (KCl), calcium chloride (CaCl_2_), and magnesium chloride (MgCl_2_), have been studied (15, 16). The use of CaCl_2_ and MgCl_2_ resulted in flavor differences compared with the full-Na Cheddar cheese. Only a mixture of NaCl and KCl achieved acceptable results (15, 16). Several studies have shown that it is possible to substitute NaCl with KCl during processing, without affecting the sensory, rheological, and stability of the Nabulsi (17), Minas (18), Halloumi (19, 20), Cheddar (21, 22), Coalho (23), Prato (24), and Sao Joao (25) cheeses, with a substitution of up to 50%. However, the higher levels of substitution can affect the flavor because of inherent off-flavors, such as bitter and metallic, making the use of flavor enhancers interesting (26).

The effects of different flavor enhancers have been evaluated in Cheddar (21, 27) and Prato cheeses (28) produced with a replacement of NaCl by KCl. Results were dependent on the flavor used, modifying the sensory attributes of cheeses in a positive or negative way. One flavor enhancer that is prominent in the international market is the yeast extract (6). The combination of peptides, amino acids, glutamic acids, and nucleotides of the yeast extracts is potentially efficient and enables labeling with natural additives (29). Shakeel-Ur-Rehman et al. (30) described a more mature flavor in reduced-fat Cheddar cheeses with the addition of yeast extract at the salting step. Silva et al. (28) revealed that the addition of yeast extract had a positive influence on the flavor of Prato cheese, minimizing the perception of bitterness resulting from the addition of KCl.

In addition to instrumental results, when modifying food composition, it is essential to evaluate the acceptability of consumers.

In Europe, to be able to name a cheese as low or very low in salt, cheese may only contain no more than 0.12 g or 0.04 g of Na, respectively, or the equivalent value for salt, per 100 g of product. To be able to name a cheese reduced in salt, it must have a minimum reduction of 25% of salt compared to its counterpart (31).

In this study, apart from reducing salt content, cheeses were lactose-free, becoming an advantage for most of the adult population. Approximately 75% of adult humans have low lactase activity, so the lactose intake can cause gastrointestinal problems, such as stomach discomfort, excessive flatulence, and soft stool or diarrhea (32). During cheese making, 90%−98% of lactose is lost in whey, and the residual lactose is fermented to lactic acid (33); therefore, cheeses with 60 days of ripening do not contain lactose. However, with shorter or medium ripening time, residual lactose could remain in cheeses. Therefore, to ensure lactose-free cheeses, lactose in cheese milk was previously hydrolyzed, obtaining lactose-hydrolyzed milk (LHM). At present, various lactose-hydrolysis technologies are available to produce LHM (34), being the most used the hydrolysis with soluble enzymes that can be performed either before or after heating (34). In this study, β-galactosidase was used during the cool storage of milk before cheese making to hydrolyze most of the lactose present in milk.

The aim of this study was to determine the effect of Na reduction and partial substitution with KCl on the physicochemical, microbiological, and sensorial characteristics and acceptability of cheese made from lactose-hydrolyzed cows' milk ripened for 30 and 60 days.

## Materials and Methods

### Cheese Making

Three independent batches of cheese were produced in the Food Technology pilot plant of the Universitat Autònoma de Barcelona (Bellaterra, Spain) using 200 L of whole pasteurized milk (72°C, 15 s) from the farm Can Badó SAT (Santa Agnès de Malanyanes, Barcelona). The day before cheese making, 0.5% of β-galactosidase (Larbus S.A., Madrid, Spain) was added to raw milk and held at 4°C for 18 h, in order to hydrolyze lactose. The additional level and time required for treating cheese milk with enzyme were chosen according to the results of preliminary experiments.

Milk was heated at 32°C, and 2% (v/v) of a lactic acid starter culture of *Lactococcus lactis* ssp. *lactis* plus *L. lactis* ssp. *cremoris* (Choozit Superstart M30; Danisco, Barcelona, Spain) was added. Subsequently, 0.01% (v/v) of CaCl_2_ (35%; Laboratorios Arroyo, Santander, Spain) and 0.03% (v/v) of commercial calf rennet (520 mg active chymosin, strength 1/10,000; Laboratorios Arroyo) were added. After 45 min, the curd was manually cut into grains of about 1 cm and heated at 36°C for 10 min to facilitate the separation of whey out of the curd. After heating, all the whey was drained off and curd was transferred to molds (1.2 kg) with a cotton cloth. Curd was pressed at 1.2 kPa for 1 h, 1.8 kPa for 1 h, 2.45 kPa for 3 h, and 3.6 kPa for 2 h with a pneumatic press (Garvía, Barcelona, Spain). Cheeses were divided into eight lots for salting with the different procedures described below. After salting, cheeses were ripened in a room at 14°C with an 85% relative humidity for 30 and 60 days, in order to obtain semi-hard and hard cheeses, respectively.

### Salting Treatments and Elaboration of Reduced-Salt Cheeses

According to Na levels, three different salting treatments of cheese were produced, namely, conventional salting for normal-Na treatment; reduced time of salting by using conventional brine of Na; and salting with brine made with a mixture of potassium (K) and Na.

A preliminary study was necessary to determine salting conditions (e.g., salting time and brining composition) to achieve a minimum of the 25% of salt reduction to comply with the denomination of reduced cheeses according to the European regulations.

Conventional salting was performed by immersion in brine (20% w/w NaCl; Sal Costa, S.A., Barcelona, Spain) at 14°C for 4.5 h in order to produce control cheeses (CN). To know the required time to achieve a minimum of the 25% of salt reduction in reduced-Na cheeses (CR), four salting times were tested (3.50, 2.50, 2, and 1.50 h).

KCl (KaliSel®, Quimidroga S.A., Barcelona, Spain) was used as a Na replacer to make substituted-Na cheeses (CS). Three different ratios of NaCl:KCl were tested (i.e., 60:40, 50:50, and 75:25) at 14°C for 4.5 h.

All cheeses were elaborated as described in the “Cheese making” section. A group of cheeses were salted in brine (20% NaCl) for 4.30 h, to be used as CN.

#### Reduced-Salt Cheeses by Reduced-Na Treatment

Three kinds of reduced-salt cheeses (CR) were salted for 2.15 h, a time chosen from previous results to obtain the necessary Na reduction. To improve the possible low salty taste in these samples, some of them were added with 0.1 and 0.2% of commercial flavor enhancers (CR0.1 and CR0.2, respectively) before salting (Table 1). The flavor enhancer was composed of commercial yeast extract (Carinsa, Barcelona, Spain), and the quantity added was chosen by the commercial specification and literature review (21, 27, 35).

**Table 1:** Denomination of cheeses.

**Cheeses**	**Flavor enhancer**	**Brining conditions**	
		**Brine**	**Time**
CN	-	20% (NaCl)	4.5 h
CR	-	20% (NaCl)	2.15 h
CR0.1	0.1 %^*a*^	20% (NaCl)	2.15 h
CR0.2	0.2 %^*b*^	20% (NaCl)	2.15 h
CS	-	20% (60 NaCl:40 KCl)	4.5 h
CS0.8	0.8 %^2^	20% (60 NaCl:40 KCl)	4.5 h
CS1.3	1.3 %^2^	20% (60 NaCl:40 KCl)	4.5 h

*CN, control cheese; CR, cheeses made with reduced-sodium treatment; CS, cheeses made with substituted-sodium treatment*.

a *Carinarome*,

b *Maxarome*.

#### Reduced-Salt Cheeses by Substituted-Na Treatment

Three batches of cheeses were immersed in brine (60% NaCl:40% KCl), and conditions were obtained from previous results. To avoid the possible bitter taste caused by KCl, two batches were previously added with 0.8% (CS0.8) and 1.3% (CS1.3) of flavor enhancer (Maxarome Select Powder AGGL, DSM, Holland) before salting (Table 1). This compound is also constituted by yeast extract rich in natural guanosine-5′-monophosphate (5′-GMP) and inosine monophosphate (IMP) and, as in the previous case, it was added at the concentrations indicated by the manufacturer and literature review (21, 28).

### Cheese Composition and Salt Content Determination

Triplicate samples were analyzed for dry matter by a gravimetric method, drying cheese samples at 102°C until constant weight (36). pH was measured with a pH meter (Crison Basic 20pH; Crison Instrument, Alella, Spain) on a ratio of cheese:distilled water (1:1) slurry. Fat content was determined by the acid butyrometric method according to the study by Van Gulik (37). Cheese nitrogen was fractionated according to the method of Kuchroo and Fox (38) to obtain the water-soluble nitrogen at pH 4.6. Total free amino acids (FAA) were determined on the water-soluble extracts by the cadmium-ninhydrin method described by Folkertsma and Fox (39) and were expressed as mg Leu for 100 g of cheese. The concentration of lactose was determined using RocheTM lactose/D-galactose enzymatic-spectrophotometric kit (R-Biopharm AG, Darmstadt, Germany).

Na and K contents were determined by inductively coupled plasma optical emission spectrometry (ICP-EOS) method using a Perkin-Elmer ICP-EOS equipment (model Optima 4300DV; Norwalk, CT, USA). Samples were previously digested in a microwave digester (Milestone Ultrawave model, Sorisole (BG), Italy) equipped with 12 Teflon vessels. A cheese sample was added with 5.0 ml of 70% ultra-pure nitric acid and digestion was carried out at 240°C for 20 min. The digested sample solution was filtered through a membrane filter (pore size 0.45 μm) and made up to 50 g with Milli-Q water. Calibration samples were prepared from single-element standard solutions (Spectrascan-Teknolab A/S-Norway) that were diluted with 10% ultra-pure nitric acid at concentrations ranging between 2 and 120 ppm for Na, and between 1 and 60 ppm for K. The general experimental conditions used were as follows: injector: alumina 2 mm ID; plasma gas flow: 15 L min^−1^; auxiliary gas flow: 0.2 L min^−1^; nebulizer gas flow: 0.75 L/min; RF power: 1,300 W; Na wavelength: 589.592 nm, and K wavelength: 766.490 nm. Results were expressed as milligram per gram of cheese.

All analyses were performed in duplicate at 30 and 60 days of ripening.

### Microbiological Analysis

Ten grams of cheese were homogenized in 90 ml of sterile peptone water (Oxoid, Hampshire, UK) for 3 min in a Stomacher (Stomacher 400 Circulator; Seward Medical, London, UK), followed by 1 min in a homogenizer (Pulsifier II®, UK). Serial dilutions were made, and microorganisms were grown in different media (Oxoid Ltd., Hampshire, UK) and incubation conditions were as follows: plate count agar (PCA) for aerobic mesophilic microorganisms incubated at 30°C for 48 h; M17 agar plus 10% lactose for lactococci incubated at 30°C for 72 h; Man, Rogosa, and Sharpe agar (MRSA) for lactobacilli incubated at 30°C for 72 h; and Violet Red Bile Glucose Agar (VRBGA) for *Enterobacteriaceae* incubated at 37°C for 24 h. Results were expressed as log colony-forming units per gram of cheese (log cfu g^−1^).

### Color Analysis

Color measurements were performed with a portable spectrocolorimeter Hunter Lab (MiniscanTM XE, Hunter Associates Laboratory, Reston, VA, USA), with a cool white fluorescent (Fcw) illuminant and observation angle of 10°. Measurements were made directly upon the samples on four different points of the surface (without rind) and the inner section of the cheeses to obtain the color space parameters, namely, L^*^ (lightness that ranges between 0 and 100), a^*^ value measures from the redness to greenness (positive to negative values, respectively), and b^*^ value measures from the yellowness to blueness (positive to negative values, respectively), according to the CIELAB scale. Measurements were made at days 30 and 60 of ripening.

### Texture Analysis

Texture profile analysis (TPA) was used to evaluate the textural properties of cheese, using a TA-TX2 Texture Analyzer (Stable Micro System Ltd., Surrey, UK) with a 245N load cell using a 35-mm diameter cylinder probe. Cube-shaped samples (1.5 cm^3^) from the internal and medium area of each cheese at each stage of ripening were cut with a device consisting of 1.5 cm parallel blades. Samples were equilibrated at room temperature (20°C) for 2 h before measurements. TPA was based on two cyclical compressions at room temperature with a test speed of 5.0 mm/s, a compression ratio of 50%, and time of 5 s between compressions. Cheeses were characterized in terms of hardness, friability, adhesiveness, springiness, cohesiveness, gumminess, and chewiness. Six replicates for each product were performed.

### Sensory Evaluation

Sensory evaluation was performed by ten panelists of the Universitat Autònoma de Barcelona who were experienced in the sensory assessment of cheeses. Each kind of cheese was compared with the CN, using a 7-point negative to positive scale (0: no differences with CN; ± 1: minimal differences with respect to the CN; ± 2: moderate differences with respect to the CN; ± 3: big differences with respect to the CN, i.e., negative or positive indicates lower or greater perception). Samples were evaluated for color (+: white. –: yellowish), odor intensity, taste (e.g., bitter, salty, acid, and after taste), and texture (e.g., hardness, springiness, adhesiveness, and friability) at 30 and 60 days of ripening. A preference test was also made on a 9-point scale from 9 (like extremely) to 1 (dislike extremely). Prior to an assessment, the outer rind of each cheese was removed, and cheeses were cut into triangular samples. The order of presentation was randomized, and cracker biscuits and mineral water at room temperature were served to assessors for palate cleansing between samples.

### Evaluation of the Consumers' Acceptability and Preference

A total of 95 and 88 consumers (for cheeses with 30 and 60 days of ripening, respectively) ranging in age between 18 and 65 years participated in the study. Participants were of both genders, namely, 36 men and 59 women for semi-hard cheeses (30 days of ripening), and 35 men and 53 women for hard cheeses (60 days of ripening).

Only cheeses that obtained the best results in the sensory evaluation were tested. Therefore, for the evaluation of acceptability for semi-hard cheeses, CR, CS, and CS0.8 were tested. For hard cheeses, consumers tasted CR, CR0.1, and CS.

To know the level of acceptability of cheeses, participants classified each cheese individually on a 9-point hedonic scale (from 1 = dislike very much to 9 = like very much). To know the level of preference, CC was also introduced and tasters classified the cheeses based on the same 9-point hedonic scale.

### Statistical Analysis

One-way analysis of variance (ANOVA) of three independent batches was performed using SPSS Statistic version 22.0 (IBM SPSS, Chicago, IL) to test differences among cheeses on each day of ripening and differences with time, with a significant level of *P* < 0.05. Mean comparisons were carried out using the Student–Newman–Keuls test.

## Results and Discussion

### Determination of Salting Conditions

According to the European Commission regulation, to be labeled as reduced in Na, cheese should have a reduction of the regular Na content of at least 25% (31). As can be seen in Table 2, to obtain reduced-salt cheeses with a reduction of Na, it was necessary for a brining time between 2 and 2.50 h. To elaborate the reduced-salt cheese with partial substitution of Na by K, the three tested conditions produced the intended reduction of Na > 25%. However, to avoid undesirable sensory modification described by several authors with a replacement of ≥ 50%, the brine with 60% NaCl:40% KCl ratio was selected.

**Table 2:** Sodium and potassium concentration of cheese.

**Cheese**	**Brining conditions**				
	**Brine**	**Time**	**Na^+^ (mg g^−1^ cheese)**	**K^+^ (mg g^−1^ cheese)**	**% Na^+^ reduction**
CN	100% NaCl	4.5 h	6.7		
CR	100% NaCl	3.5 h	6.5		3
CR	100% NaCl	2.5 h	5.2		22.5
CR	100% NaCl	2 h	4.4		33.9
CR	100% NaCl	1.5 h	4.05		39.6
CS	60%NaCl:40%KCl	4.5 h	3.3	4.2	50.7
CS	50%NaCl:50%KCl	4.5 h	3.1	5.3	53.7
CS	25%NaCl:75%KCl	4.5 h	1.7	6.7	74.6

*CN, control cheese; CR, cheeses made with reduced-sodium treatment; CS, cheeses made with substituted-sodium treatment*.

### Composition of Cheeses

All cheeses could be described as reduced in salt, showing >25% of Na reduction compared to CC (Tables 3, 4).

**Table 3:** Composition of reduced-sodium cheeses (CR) at 30 and 60 days of ripening.

Cheeses*	**Days of ripening**	**pH**	**Dry matter (%)**	**Na^+^ (mg g^−1^ cheese)**	**% Na reduction**	**FAA (mg Leu 100 g^−1^ cheese)**
CN	30	4.87 ± 0.04^Ab^	53.84 ± 0.67^Aa^	7.21 ± 0.02^A^		2.06 ± 1.00^Aa^
CR	30	4.81 ± 0.02^Ab^	53.85 ± 0.67^Aa^	5.01 ± 0.03^B^	30.5	2.27 ± 1.60^Aa^
CR0.1	30	4.81 ± 0.03^Ab^	54.33 ± 1.01^Aa^	5.09 ± 0.04^B^	29.4	2.60 ± 1.56^Aa^
CR0.2	30	4.84 ± 0.01^Ab^	53.65 ± 0.25^Aa^	5.07 ± 0.04^B^	29,6	2.65 ± 1.20^Aa^
CN	60	4.75 ± 0.03^Aa^	58.27 ± 0.83^Ab^	8.53 ± 0.02^A^		3.46 ± 0.21^Ab^
CR	60	4.74 ± 0.04^Aa^	58.91 ± 0.28^ABb^	5.97 ± 0.02^B^	30	3.41 ± 0.46^Ab^
CR0.1	60	4.77 ± 0.02^Aa^	59.48 ± 0.52^BCb^	5.76 ± 0.05^B^	32.5	4.20 ± 0.34^Bb^
CR0.2	60	4.73 ± 0.03^Aa^	60.05 ± 0.85^Cb^	5.71 ± 0.04^B^	33	4.19 ± 0.28^Bb^

AB *Means for the same parameter and day of ripening with different superscripts differ (P ≤ 0.05)*.

ab *Means for the same cheese and different day of ripening with different superscripts differ (P ≤ 0.05)*.

* *CN, Control cheeses; CR, reduced-sodium cheeses by reduced-sodium treatment; CR0.1, reduced-sodium cheeses by reduced-sodium treatment with 0.1% of flavor enhancer; CR0.2, reduced-sodium cheeses by reduced-sodium treatment with 0.2% of flavor enhancer*.

**Table 4:** Composition of substituted-sodium cheeses (CS) at 30 and 60 of ripening.

**Cheeses**	**Days of ripening**	**pH**	**Dry matter (%)**	**Na^+^ (mg g^−1^ cheese)**	**% Na reduction**	**FAA (mg Leu 100 g^−1^ cheese)**
CN	30	4.83 ± 0.03^Aa^	53.09 ± 2.03^Aa^	7.33 ± 0.03^A^		4.10 ± 0.10^Aa^
CS	30	4.90 ± 0.06^Ba^	52.32 ± 0.94^Aa^	5.53 ± 0.04^B^	24.6	4.60± 0.03^Ba^
CS0.8	30	4.86 ± 0.01^ABa^	53.02 ± 1.63^Aa^	5.4 ± 0.04^B^	26.3	4.83 ± 0.06^Ca^
CS1.3	30	4.86 ± 0.02^ABa^	52.43 ± 0.26^Aa^	5.28 ± 0.04^B^	28	4.87 ± 0.27^Ca^
CN	60	4.82 ± 0.01^Aa^	57.05 ± 0.67^Ab^	8.66 ± 0.02^A^		5.15 ± 1.16^Ab^
CS	60	4.85 ± 0.03^Aa^	57.19 ± 0.72^Ab^	5.37 ± 0.04^B^	38	5.74 ± 0.80^ABb^
CS0.8	60	4.87 ± 0.06^Aa^	57.22 ± 0.72^Ab^	5.97 ± 0.05^B^	31.1	6.52 ± 1.17^BCbc^
CS1.3	60	4.88 ± 0.09^Aa^	57.79 ± 1.05^Ab^	5.69 ± 0.06^B^	31.7	7.18 ± 0.48^Cb^

AB *Means for the same parameter and day of ripening with different superscripts differ (P ≤ 0.05)*.

ab *Means for the same cheese and different day of ripening with different superscripts differ (P ≤ 0.05)*.

* *CN, Control cheeses; CS, reduced-sodium cheeses by substituted-sodium treatment; CS0.8, reduced-sodium cheeses by substituted-sodium treatment with 0.8% of flavor enhancer; CS1.3, reduced-sodium cheeses by substituted-sodium treatment with 1.3% of flavor enhancer*.

A slight pH reduction was observed with ripening time, due to the fermentation process. The reduction of salt by the reduction of brining time treatment did not affect the pH of cheeses (Table 3). These results are in agreement with other authors who did not find a significant effect of salting conditions on the pH of Cheddar (9) or Mozzarella cheeses (9, 40) with reductions of up to 60% of salt.

The partial replacement of NaCl by KCl produced a slight increase in the pH levels in cheeses at 30 days of ripening (Table 4). Similar results were described in Halloumi (19, 20, 41, 42) and Mozzarella cheeses (43), and it was attributed to the higher pH of KCl-NaCl brines. Fitzgerald and Buckley (15) also described higher pH in Cheddar cheeses salted by KCl compared to those salted by NaCl, CaCl_2_ or MgCl_2_. At 60 days of ripening, no differences in pH values were observed, neither with the ripening time, showing that at 30 days of ripening, the transformation of sugars into lactic acid was practically complete.

Dry matter content was not affected by the reduction of salt or by the partial substitution of NaCl by KCl in cheeses with 30 days of ripening (Tables 3, 4). Baptista et al. (44) also found no differences in dry matter content in Prato cheese with a reduction of 25% and 50% of salt at 15 days of ripening. Neither differences were observed in Halloumi (41) or Mozzarella cheese (45) with partial substitution of NaCl for KCl. Ripening time produced an increase in dry matter content due to water evaporation. At 60 days of ripening, a slight increase in dry matter was observed for reduced-NaCl cheeses, only significant in CR cheeses containing flavor enhancer. Possibly, the addition of enhancer as powder could produce the increase of dry matter in cheeses.

Proteolysis is the most important biochemical event that occurs during the ripening of cheeses (46), and it plays a direct role on cheese flavor and texture development in most cheese varieties (47). Salt, along with other parameters, influences the rate of proteolysis, so it is important to study it in salt-reduced cheeses. According to the literature, it has been generally informed that an increase in proteolysis in cheeses with the reduction of salt concentration (48) attributed to the increase in water activity and the reduction in NaCl bacteriostatic effect. In CR, the levels of FAA, an indicator of secondary proteolysis, were higher in cheeses containing flavor enhancer, although differences were only statistically significant in cheeses at 60 days of ripening (Table 3). However, no significant differences were found between CR and CN. Baptista et al. (44) also found no differences in the development of proteolysis due to a reduction of salt in Prato cheese. Contrarily, Dugat-Bony et al. (49) described an increased secondary proteolysis in semi-hard cheeses reduced in salt, and Murtaza et al. (13) also found an increase in the FAA in buffalo milk Cheddar cheese with the reduction of salt content. However, Møller et al. (50) and McCarthy et al. (51) described more FAA in high-salt Cheddar cheeses than in reduced cheeses. Gandhi and Shah (52) observed an increase of amino acids during storage in Akawi cheeses with a reduction from 10 to 7.5% NaCl in brine solution; however, this did not happen with a higher reduction (up to 5% NaCl in brine solution). These differences between cheese varieties could be attributed to variances in cheese making, which affect differently the growth of microorganisms, and several parameters influence the activity of their proteolytic enzymes, which are responsible for FAA release.

The partial substitution of NaCl by KCl increased the FAA levels, being higher in cheeses containing flavor enhancer (Table 4). These results are in agreement with Silva et al. (35) who found higher proteolysis values in reduced-Na Prato cheeses (50% NaCl:50% KCl) than in conventional cheeses, showing the highest values of the reduced cheeses with flavor enhancers. In contrast, no significant differences in secondary proteolysis have been reported with the partial substitution of NaCl by KCl in Halloumi (19), Prato (24), semi-hard (49), Feta (53), Cheddar (22), or Akawi cheeses (54). As expected, all cheeses increased the levels of FAA with ripening time because of the activity of proteolytic enzymes. Cheeses containing flavor enhancer presented the highest level of FFA, since a mixture of peptides and amino acids composes the yeast extract.

### Microbiology

Salt reduction slightly increased the lactic microbial counts in cheeses (Table 5). An increase in the starter bacteria with the reduction of salt was also found in Cheddar (14, 55) and Akawi (52) cheeses. Sheibani et al. (56) did not find differences in the growth of starter culture in hard-type cheeses reduced at 17% and 35% of salt; however, a reduction at 50% of salt increased their growth. Similarly, the reduction of salt did not modify the total bacteria counts in Sao Joao cheeses (25). The addition of flavor enhancer significantly decreased the microorganism counts in CR, observing the lowest values in CR0.2 at 60 days of ripening. These results are not consistent with those of Shakeel-Ur-Rehmnan et al. (30) who observed an increase in non-starter lactic bacteria (NSLAB) at the early stages of ripening in Cheddar cheese with the incorporation of yeast extract, nor by the Silva et al. (35) who described significantly lower lactic acid bacteria counts in reduced-Na Prato cheese, with and without flavor enhancer.

**Table 5:** Microbiological results (log cfu g^−1^ cheese) of reduced-sodium cheeses by reduced-sodium treatment (CR) at 30 and 60 days of ripening.

Cheeses*	**Days of ripening**	**Total counts**	**Lactococci**	**Lactobacilli**
CN	30	8.60 ± 0.11^BCb^	8.55 ± 0.03^ABb^	8.63 ± 0.23^Bb^
CR	30	9.01 ± 0.51^Cb^	9.04 ± 0.39^Bb^	9.11 ± 0.37^Cb^
CR0.1	30	8.26 ± 0.39^ABb^	8.37 ± 0.47^Ab^	8.16 ± 0.12^Ab^
CR0.2	30	7.74 ± 0.06^Ab^	8.04 ± 0.06^Ab^	8.00 ± 0.00^Ab^
CN	60	6.95 ± 0.07^Bca^	7.76 ± 0.59^Ba^	7.26 ± 0.21^Ba^
CR	60	7.09 ± 0.12^Ca^	7.82 ± 0.01^Ba^	7.23 ± 0.07^Ba^
CR0.1	60	6.81 ± 0.05^Ba^	6.99 ± 0.12^ABa^	7.07 ± 0.10^Ba^
CR0.2	60	6.00 ± 0.00^Aa^	6.75 ± 0.21^Aa^	6.59 ± 0.16^Aa^

AB *Means for the same parameter and day of ripening with different superscripts differ (P ≤ 0.05)*.

ab *Means for the same cheese and different day of ripening with different superscripts differ (P ≤ 0.05)*.

* *CN, Control cheeses; CR, reduced-sodium cheeses by reduced-sodium treatment; CR0.1, reduced-sodium cheeses by reduced-sodium treatment with 0.1% of flavor enhancer; CR0.2, reduced-sodium cheeses by reduced-sodium treatment with 0.2% of flavor enhancer*.

Microbial counts decreased with time from 30 to 60 days of ripening because of the appearance of unfavorable conditions for the development of microorganisms throughout the ripening time. No *Enterobacteriaceae* was observed in any cheese at 30 or 60 days of ripening.

The partial substitution of NaCl by KCl did not produce significant differences in total and lactic microbial growth (Table 6). These results are in agreement with those of Dugat-Bony et al. (49) in semi-hard cheese and Ayyash & Shah (41) and Kamleh et al. (20) in Halloumi cheese. The addition of flavor enhancer did not cause any effect on lactic microbial growth. Silva et al. (35) described a slight decrease of the starter bacteria in Prato cheeses elaborated with a ratio of NaCl:KCl (1:1) at 30 and 60 days of ripening. However, the addition of yeast extract did not produce any effect in salt-reduced Prato cheeses. Microbial counts decreased from 30 to 60 days of ripening. An average of 2.65 log cfu g^−1^ of *Enterobacteriaceae* was found in cheeses with 30 days of ripening, due to the environmental contamination and the manipulation during cheese making. No significant differences were observed between cheeses. These numbers decreased until non-detectable levels at 60 days.

**Table 6:** Microbiological results (log cfu g^−1^ cheese) of reduced-sodium cheeses by substituted-sodium treatment (CS) at 30 and 60 days of ripening.

Cheeses*	**Days of ripening**	**Total counts**	**Lactococci**	**Lactobacilli**	***Enterobacteriaceae***
CN	30	8.53 ± 0.38^Ab^	8.65 ± 0.29^Ab^	8.60 ± 0.35^Ab^	2.75 ± 0.17
CS	30	8.40 ± 0.31^Ab^	8.40 ± 0.37^Ab^	8.50 ± 0.35^Ab^	2.68 ± 0.10
CS0.8	30	8.20 ± 0.37^Ab^	8.33 ± 0.72^Ab^	8.48 ± 0.49^Ab^	2.70 ± 0.33
CS1.3	30	8.18 ± 0.44^Ab^	8.23 ± 0.24^Ab^	8.48 ± 0.37^Ab^	2.48 ± 0.17
CN	60	7.05 ± 0.47^Aa^	7.13 ± 0.32^Aa^	7.23 ± 0.38^Aa^	ND
CS	60	7.30 ± 0.27^Aa^	7.08 ± 0.59^Aa^	7.13 ± 0.42^Aa^	ND
CS0.8	60	7.15 ± 0.21^Aa^	7.40 ± 0.24^Aa^	7.48 ± 0.29^Aa^	ND
CS1.3	60	7.53 ± 0.40^Aa^	7.73 ± 0.84^Aa^	7.60 ± 0.58^Aa^	ND

AB *Means for the same parameter and day of ripening with different superscripts differ (P ≤ 0.05)*.

ab *Means for the same cheese and different day of ripening with different superscripts differ (P ≤ 0.05)*.

* *CN, Control cheeses; CS, reduced-sodium cheeses by substituted-sodium treatment; CS0.8, reduced-sodium cheeses by substituted-sodium treatment with 0.8% of flavor enhancer; CS1.3, reduced-sodium cheeses by substituted-sodium treatment with 1.3% of flavor enhancer*.

*ND, non detected*.

### Color and Texture

Texture of cheese is one of the most important parameters for consumers, which could be altered by the reduction of Na or the replacement of Na by K, thus affecting its acceptability.

No significant differences were found in texture parameters among cheeses reduced in salt at the same storage day. Hardness, fracturability, gumminess, and chewiness increased from 30 to 60 days of ripening, whereas cohesiveness decreased. No differences were observed in adhesiveness or springiness over time (results not shown).

Sheibani et al. (56) described that the reduction of salt decreases hardness and gumminess of hard-type cheeses. Murtaza et al. (13) and Ganesan et al. (9) also described a reduction of hardness and cohesiveness in Cheddar cheese reduced in salt.

**Figure 1:**
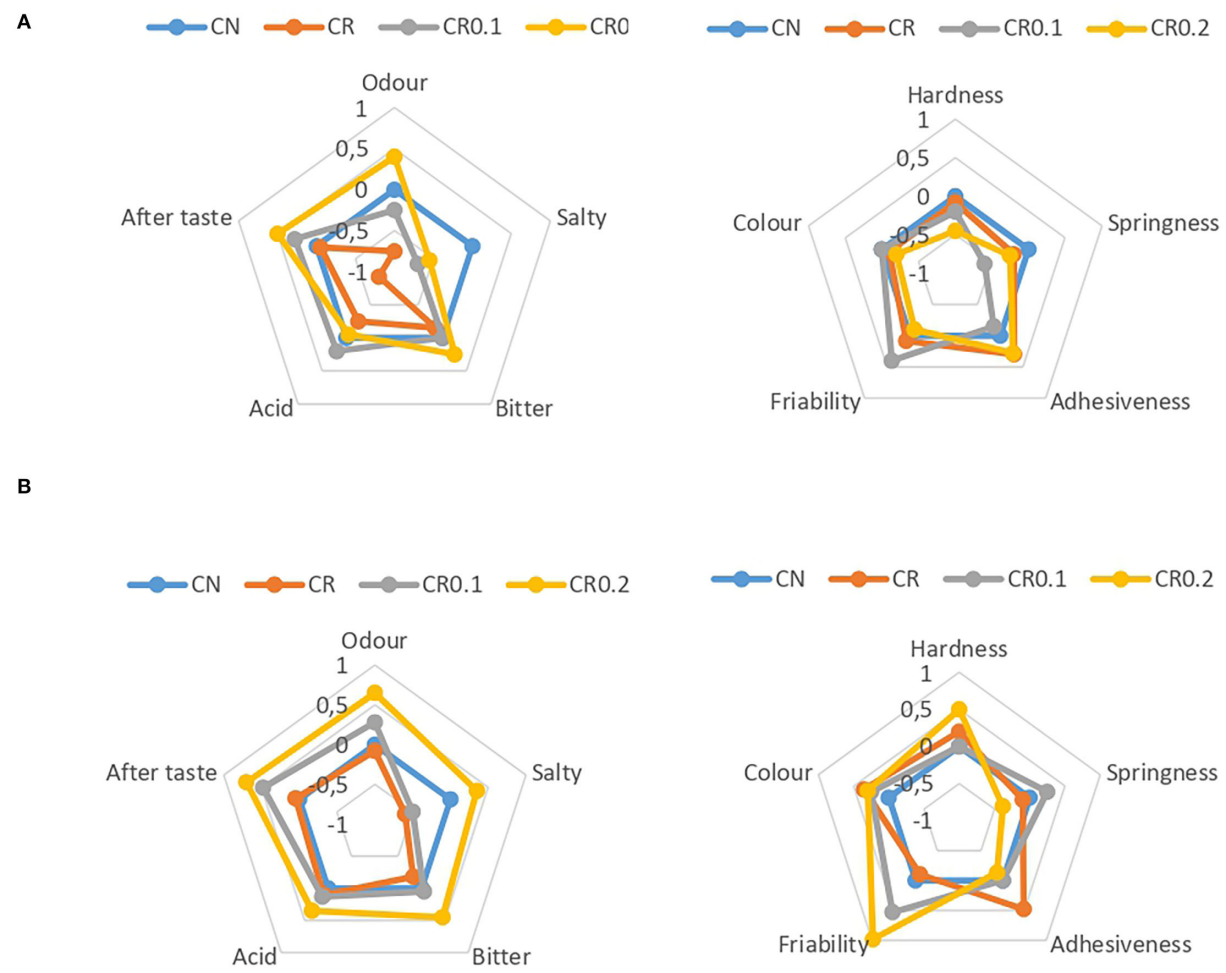
Results of sensorial analysis of reduced-sodium (Na) cheeses by reduced-Na treatment (CR) at 30 days **(A)** and 60 days **(B)** of ripening.

The reduction of Na did not affect the color of cheeses (Table 7). Only the higher percentage of flavor enhancer produced an increase in lightness and a decrease in the b^*^ parameter, giving less yellowish cheeses. The flavor enhancer used had a strong brown color that could have changed the color of cheeses. Nevertheless, the panelists did not detect these differences (Figure 1). The lightness decreased, and a^*^ and b^*^ parameters increased with time.

**Table 7:** Color results of reduced-sodium cheeses by reduced-sodium treatment (CR) at 30 and 60 days of ripening.

Cheeses*	**Days of ripening**	L*	a*	b*
CN	30	91.47 ± 0.62^Ab^	−2.10 ± 0.28^Aa^	15.57 ± 0.44^Aa^
CR	30	91.72 ± 0.58^ABb^	−2.00 ± 0.10^Aa^	15.39 ± 0.41^Aa^
CR0.1	30	91.20 ± 0.73^Ab^	−2.16 ± 0.32^Aa^	15.68 ± 0.35^Aa^
CR0.2	30	92.26 ± 0.35^Bb^	−2.15 ± 0.02^Aa^	14.36 ± 0.39^Ba^
CN	60	89.85 ± 1.27^Aa^	−1.74 ± 0.19^Ab^	16.93 ± 0.41^ABb^
CR	60	89.98 ± 0.31^ABa^	−1.70 ± 0.14^Ab^	17.12 ± 0.63^Bb^
CR0.1	60	89.58 ± 1.06^Aa^	−1.23 ± 1.06^Ab^	16.50 ± 0.64^ABb^
CR0.2	60	90.86 ± 0.43^Ba^	−1.68 ± 0.34^Ab^	16.27 ± 1.02^Ab^

AB *Means for the same parameter and day of ripening with different superscripts differ (P ≤ 0.05)*.

ab *Means for the same cheese and different day of ripening with different superscripts differ (P ≤ 0.05)*.

* *CN, Control cheeses, CR, reduced-sodium cheeses by reduced-sodium treatment, CR0.1, reduced-sodium cheeses by reduced-sodium treatment with 0.1% of flavor enhancer; CR0.2, reduced-sodium cheeses by reduced-sodium treatment with 0.2% of flavor enhancer*.

The partial substitution of NaCl by KCl did not affect the texture profile of cheeses. Only hardness increased in all cheeses with time, while the rest of the texture parameters remained unchanged from 30 to 60 days of ripening (results not shown). These results are consistent with other studies with NaCl reduction using KCl in Kefalograviera (53), Nabulsi (17), Akawi (54), Halloumi (19), and Prato cheeses (24).

**Figure 2:**
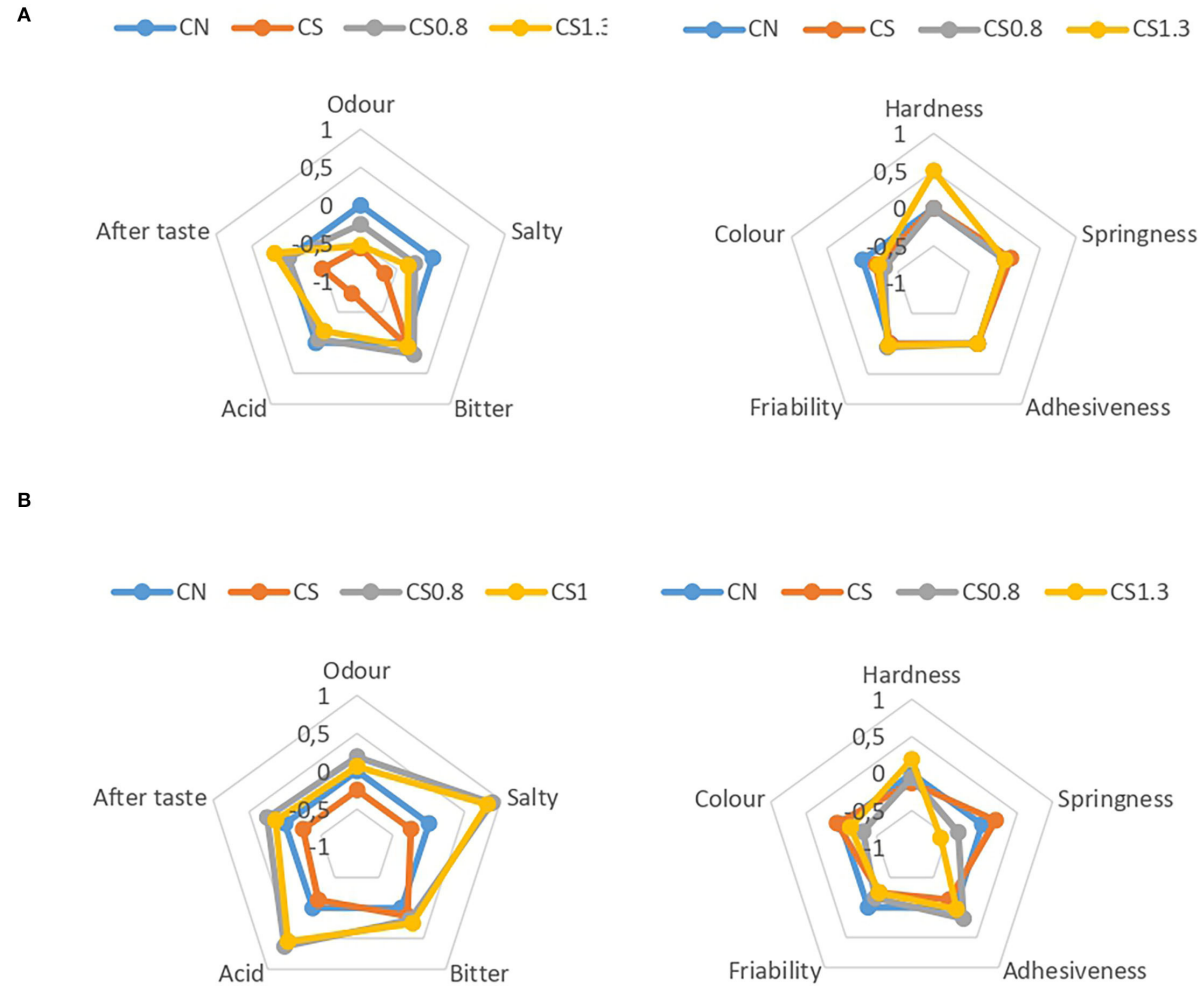
Results of sensorial analysis of reduced-Na cheeses by substituted-Na treatment (CS) at 30 days **(A)** and 60 days **(B)** of ripening.

Substituted cheeses presented a slight increase of lightness at 30 days of ripening, only statistically significant in cheeses with a higher amount of flavor enhancer (CP1.3). No differences were found for a^*^ and b^*^ parameters (Table 8). At 60 days of ripening, cheeses were similar to color parameters, and only cheeses containing flavor enhancer presented higher and lower a^*^ and b^*^ parameters, respectively, leading to redder and less yellow cheeses. This trend could be explained by the strong brown color of the flavor enhancer. However, the panelists did not appreciate these instrumental color differences (Figure 2). Lightness and a^*^ parameter decreased with time, meanwhile b^*^ parameter increased from 30 to 60 days of ripening, coinciding with the results of Duffose et al. (57), Pinho et al. (58), Juan et al. (59), Gheisari et al. (60), and Alvarez & Fresno (61) who described the effects of ripening time in the color parameters of cheeses.

**Table 8:** Color results of reduced-sodium cheeses by substituted-sodium treatment (CS) at 30 and 60 days of ripening.

Cheeses*	**Days of ripening**	L*	a*	b*
CN	30	90.67 ± 1.14^Aa^	1.76 ± 0.18^Ab^	14.86 ± 0.72 ^Aa^
CS	30	90.87 ± 0.58^ABb^	1.75 ± 0.16^Ab^	15.01 ± 0.53 ^Aa^
CS0.8	30	91.19 ± 1.07^ABb^	1.70 ± 0.26^Aa^	14.38 ± 0.70 ^Aa^
CS1.3	30	91.54 ± 0.55^Bb^	1.74 ± 0.25^Aa^	14.73 ± 0.77 ^Aa^
CN	60	90.01 ± 0.81^Aa^	1.42 ± 0.10^Aa^	15.86 ± 0.54 ^Bb^
CS	60	89.77 ± 1.24^Aa^	1.42 ± 0.06^Aa^	15.50 ± 0.68 ^ABa^
CS0.8	60	90.38 ± 0.92^Aa^	1.53 ± 0.13^Ba^	15.13 ± 0.87 ^Ab^
CS1.3	60	90.04 ± 0.88^Aa^	1.56 ± 0.15^Ba^	15.18 ± 0.42 ^Aa^

AB *Means for the same parameter and day of ripening with different superscripts differ (P ≤ 0.05)*.

ab *Means for the same cheese and different day of ripening with different superscripts differ (P ≤ 0.05)*.

* *CN, Control cheeses; CS, reduced-sodium cheeses by substituted-sodium treatment; CS0.8, reduced-sodium cheeses by substituted-sodium treatment with 0.8% of flavor enhancer; CS1.3, reduced-sodium cheeses by substituted-sodium treatment with 1.3% of flavor enhancer*.

### Sensory Evaluation

Figure 1 shows the sensory results of CR at 30 and 60 days of ripening. We must take into consideration that CR were compared to CCs, with minimal, moderate, and high differences being valued in the scale of 1–3, respectively. The reduction of salt had no significant difference in the color or texture perception of cheeses. The aroma was higher in cheese containing 0.2% of flavor enhancer, with minimal differences with respect to CC. The reduction of salt caused less sensation of saltiness in cheeses, becoming CR the least valued, followed by C0.1 and C0.2. However, at 60 days of ripening, cheeses containing 0.2% of flavor enhancer were reported as the saltiest. These cheeses were also valued as the most bitter with the greatest aftertaste. Differences, although statistically significant, were always <1, being classified as minimal.

At 30 and 60 days of ripening, CCs were preferred by the panelists, showing scores of 5.73 and 6.75, respectively. At 30 days of ripening, salt-reduced cheese without flavor enhancer was preferred to cheeses containing this compound, with values of 5.07, 4.67, and 4.87 in CR, CR0.1, and CR0.2, respectively. However, at 60 days of ripening, C0.1 (6.06) was preferred to CR (5.75), showing that the addition of 0.1% of this flavor enhancer could improve the sensorial characteristics of CR. CN and CR0.1 were valued with a score of >6, described as a “slightly like them.” In contrast, the addition of 0.2% of flavor enhancer reduced the preferences of cheeses (5.38), possibly due to the increase in salty, bitter, and aftertaste. These results indicate that the addition of 0.1% of flavor enhancer improves the sensorial perception of hard CR. However, it would not be a good alternative to semi-hard CR.

The partial substitution of NaCl by KCl had no significant difference in color, texture, or aroma of cheeses (Figure 2). The reduction of salt caused less sensation of saltiness in cheeses at 30 days of ripening. However, at 60 days, cheeses containing flavor enhancer presented a higher salty taste than CN or CS cheeses. The addition of flavor enhancer also slightly increased the acid taste at 60 days of ripening.

At 30 days of ripening, panelists preferred CCs, with a medium value of 6.54. The substitution of NaCl by KCl reduced the preferences of cheeses, with values between 5 and 6, which means that “neither like nor dislike”, having the higher value of CS0.8 cheese (5.95) followed by CS1.3 (5.80) and CS (5.70). The addition of 0.8% of flavor enhancer appears to slightly improve cheese's preference. It seems that sensory differences due to the substitution of NaCl by KCl decreased with ripening time, observing better scores in cheeses with 60 days. The mean of the preference values of CN (6.63), CS (6.31), CS0.8 (6.05), and CS1.3 (5.99) was not statistically different. No significant differences in the sensory attributes score between CN and cheeses made with a mixture of NaCl/KCl were found in Akawi (54), Kefalograviera (53), and semi-hard cheeses (49). Kamleh et al. (20) also did not find differences between CN and Halloumi cheeses with a mixture of NaCl/KCl; however, bitterness was higher in the latest.

### Consumers' Acceptability

To know the general acceptability of consumers, untrained panelists tasted the cheeses with the best sensorial scores in the sensorial analysis. In this sense, for semi-hard cheeses, consumers evaluated CR, CS, and CS0.8 cheeses. There were no significant differences between cheeses reduced in salt by the reduction of NaCl or by KCl substitution, showing the viability of both strategies to produce cheeses reduced in salt (Table 9). Consumers showed a similar preference for both reduced cheeses and conventional cheeses. However, the addition of flavor enhancer decreased the acceptability and preference of cheeses, concluding that it would not be appropriate to add it under these conditions. Baptista et al. (44) did not observe differences in the acceptability of Prato cheeses with a reduction of 25% and 50% of salt with 30 days of ripening. Costa et al. (24) did not find differences in the acceptability of Prato cheese with a replacement of 40% of Na by KCl stored for 30 days. The replacement of up to 50% salt substitution also did not alter the acceptability of Coalho cheeses stored for 30 days (23).

**Table 9:** Consumer acceptance and preference of cheeses with 30 and 60 days of ripening.

Cheeses*	**Acceptance**		**Preference**	
	**30 days**	**60 days**	**30 days**	**60 days**
CN			6.49 ± 1.66 ^a^	7.22 ± 1.31 ^a^
CR	7.04 ± 1.07 ^a^	7.19 ± 1.17 ^a^	6.28 ± 1.57 ^a^	6.61 ± 1.18 ^b^
CS	6.84 ± 1.28 ^a^	6.46 ± 1.44 ^b^	6.09 ± 1.73 ^ab^	6.22 ± 1.31 ^b^
CR0.1		5.82 ± 1.36 ^c^		5.60 ± 1.59 ^c^
CS0.8	5.90 ± 1.62 ^b^		5.56 ± 1.65 ^b^	

ab *Means for the same day of ripening with different superscripts differ (P ≤ 0.05). Based on a 9-point hedonic scale (1, dislike extremely; 5, neither like nor dislike; 9, like extremely)*.

*CN, control cheeses; CR, reduced-sodium cheeses by reduced-sodium treatment; CS, reduced-sodium cheeses by substituted-sodium treatment; CR0.1, reduced-sodium cheeses by reduced-sodium treatment with 0.1% of flavor enhancer; CS0.8, reduced-sodium cheeses by substituted-sodium treatment with 0.8% of flavor enhancer*.

For cheeses with 60 days of ripening, consumers evaluated CR, CR0.1, and CS cheeses. The acceptability of CR was higher than CS (Table 9). Consumer comments highlighted the pleasant flavor of CR cheeses. Lindsay et al. (62) also described a lower preference score for reduced Cheddar cheeses made with a ratio of NaCl:KCl (1:1) than those produced with a reduction of NaCl. In our study, the preference of both was lower than CC. As in the previous evaluation, the addition of flavor enhancer decreased the acceptability and preference of cheeses.

The addition of flavor enhancers in cheeses elaborated with mixtures of 40% NaCl:60% KCl ratio was studied by Grummer et al. (21), describing that some seem to affect positively, whereas others have a negative effect on consumers' acceptability of Cheddar cheeses. Silva et al. (28) described that the addition of 1% of yeast extract in substituted-Na Prato cheese increased the salty taste, reduced the typical flavor, and minimized the perception of unpleasant taste.

A possible hypothesis to explain the lower acceptability found in our cheeses added with yeast extract could be associated with the development of flavor compounds from the amino acids generated by the NSLAB. Cheeses with flavor enhancer had higher FAA levels than the rest of the cheeses (Tables 3, 4); however, further studies are needed to evaluate free fatty acids and FAA profiles and the volatile compounds of these cheeses. Future research should be carried out by testing different percentages of yeast extract and/or with different flavor enhancers, in order to improve the obtained results.

## Conclusion

The reduction of NaCl concentration or partial substitution by KCl (a ratio of 60 NaCl:40 KCl) could be a good strategy to produce hard and semi-hard lactose-free cow's milk cheeses reduced in salt. Both methodologies allow the production of healthier and sensorially acceptable cheeses, without significant modification of pH, dry matter, proteolysis, microbial growth, texture, and color of cheeses. The addition of the yeast extract as a flavor enhancer in the tested conditions increased FAAs levels and the salty, bitter, and aftertaste of cheeses, reducing their acceptability. A larger study with different percentages of flavor enhancer could improve the sensorial and preferential results obtained.

## Data Availability Statement

The original contributions presented in the study are included in the article/supplementary material, further inquiries can be directed to the corresponding author.

## Author Contributions

BJ analyzed the data and wrote the manuscript. A-JT and VF critically reviewed the article. All authors contribute to the study concept and supervision of cheese production and analysis. All authors gave final approval for all aspects of the work, agreed to be fully accountable for ensuring the integrity and accuracy of the work, and read and approved the final manuscript.

## Conflict of Interest

The authors declare that the research was conducted in the absence of any commercial or financial relationships that could be construed as a potential conflict of interest.

## Publisher's Note

All claims expressed in this article are solely those of the authors and do not necessarily represent those of their affiliated organizations, or those of the publisher, the editors and the reviewers. Any product that may be evaluated in this article, or claim that may be made by its manufacturer, is not guaranteed or endorsed by the publisher.
